# Lipschütz Ulcer: An Unusual Diagnosis that Should Not be Neglected

**DOI:** 10.1055/s-0041-1729147

**Published:** 2021-06-02

**Authors:** Daniela Alexandra Gonçalves Pereira, Eliana Patrícia Pereira Teixeira, Ana Cláudia Martins Lopes, Ricardo José Pina Sarmento, Ana Paula Calado Lopes

**Affiliations:** 1 Centro Hospitalar Barreiro-Montijo, Barreiro, Portugal

**Keywords:** vulvar ulcer, adolescent, non-venereal lesions

## Abstract

The diagnosis of genital ulcers remains a challenge in clinical practice. Lipschütz ulcer is a non-sexually transmitted rare and, probably, underdiagnosed condition, characterized by the sudden onset of vulvar edema along with painful necrotic ulcerations. Despite its unknown incidence, this seems to be an uncommon entity, with sparse cases reported in the literature. We report the case of an 11-year-old girl who presented at the emergency department with vulvar ulcers. She denied any sexual intercourse. The investigation excluded sexually transmitted infections, so, knowledge of different etiologies of non-venereal ulcers became essential. The differential diagnoses are extensive and include inflammatory processes, drug reactions, trauma, and malignant tumors. Lipschütz ulcer is a diagnosis of exclusion. With the presentation of this case report, the authors aim to describe the etiology, clinical course, and outcomes of this rare disease, to allow differential diagnosis of genital ulceration.

## Introduction

The differential diagnosis of genital ulcers is challenging in clinical practice. Overall, the etiology of vulvar ulcers is commonly infectious; nonetheless, they may be a presentation of a wide variety of pathologies, such as autoimmune disorders, inflammatory processes, drug reactions, trauma, or malignant tumors.

Vulvar ulcers are unusual in non-sexually active adolescent girls. When diagnosed in young girls, parents and clinicians should inicially suspect sexually transmitted infection due to sexual contact or abuse. After excluding this scenario, knowledge of different etiologies of non-venereal ulcers becomes essential, considering the pathological characteristics, related symptoms, and clinical manifestations.

In 1913, the Austrian dermatologist Benjamin Lipschütz 1 first described acute genital ulcers in adolescent girls without any evidence of sexually transmitted infections. These ulcers, named Lipschütz ulcers (LUs), are also known as acute genital ulcers, reactive non-sexually related acute genital ulcers, ulcus vulvae acutum, acute vulval ulcers, or primary aphthous ulcers.

The correct incidence of this disease cannot be estimated because it continues being poorly understood and underdiagnosed. The absence of established diagnostic criteria turns this entity into a diagnosis of exclusion.

The aim of the present case report is to describe the etiology, clinical course, and outcomes of LUs allowing the performance of a correct differential diagnosis of vulvar ulcerations.

## Case Report

An 11-year-old girl, healthy and without any comorbidities, presented at our emergency department with painful vulvar lesions, vulvar edema, and burning sensation on urination. The symptoms started 6 days before and were preceded by a sudden onset of fever, malaise, and odynophagie. She denied any sexual intercourse for the time being, trauma, oral ulcerations, drugs intake, recent travels, or similar previous episodes.

**Fig. 1:**
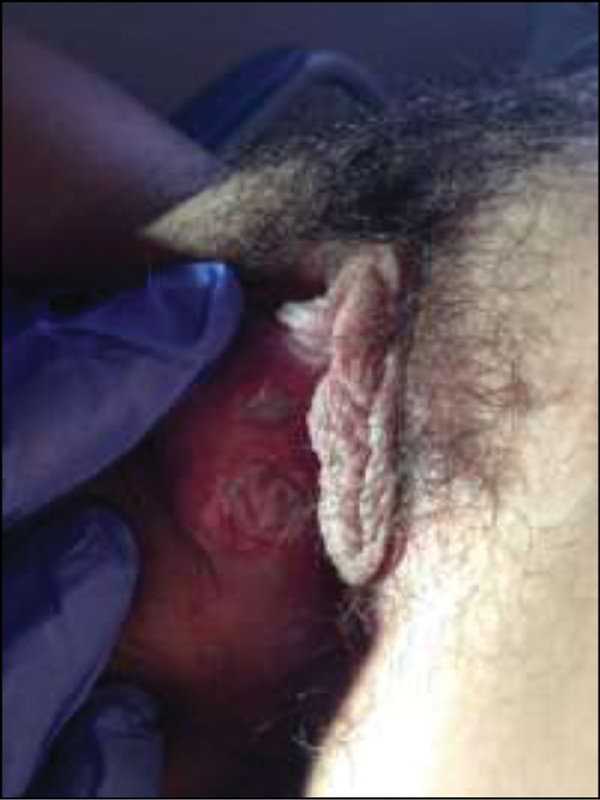
Day 6.

Physical examination revealed three painful ulcerated and necrotic lesions on the medial face of the right labia minora, the largest with 15 mm in diameter, with regular and well delimited margins, an overlying grey exudate surrounded by a purpuric halo ( Figure 1 ). No enlarged lymph nodes were detected, neither other skin or mucous membrane lesions.

In the laboratory evaluation, despite the total leukocyte count being normal, there was mild lymphocytosis (50%). C-reactive protein and liver enzymes where within the normal range. Herpes simplex virus, cytomegalovirus, treponema pallidum, hepatitis C virus, clamydia trachomatis, toxoplasmosis, mycoplasma, and human immunodeficiency virus serologies were all negative. There was evidence of prior Epstein-Barr virus infection (IgG positive, IgM negative).

**Fig. 2:**
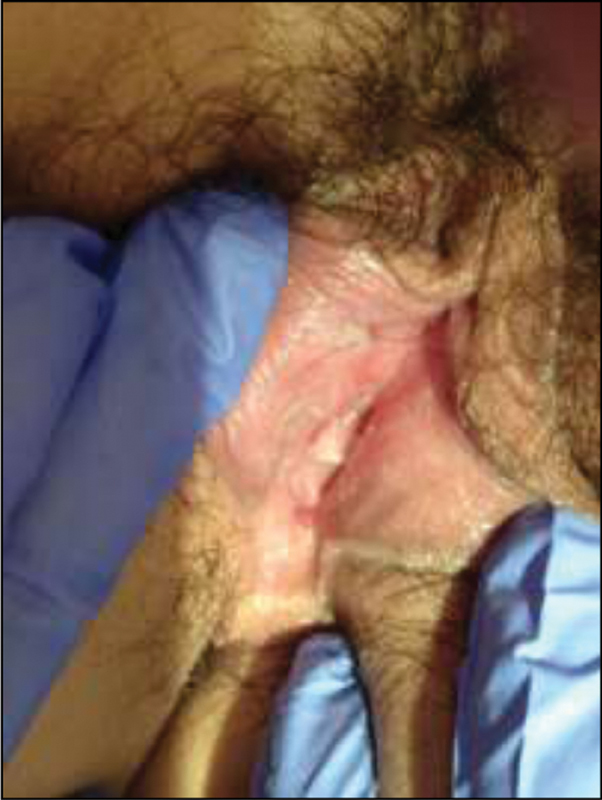
Day 14.

The patient was treated with topical cinchocaine 10 mg/g, for pain relief, and comfort measures (sitz baths and voiding in the tub to minimize external dysuria), with progressive health improvement ( Figure 2 ). Complete healing occurred in about 3 weeks, with no scarring and no recurrence to date.

## Discussion

Lipschütz ulcers are a rare cause of acute vulvar ulcerations of nonvenereal origin and in most cases affect young people without previous history of sexual contact. This condition presents with sudden onset of a painful vulvar ulcer, that is usually preceded by influenza or mononucleosis-like symptoms, such as malaise, fever, asthenia, myalgia, pharyngotonsilitis, lymphadenopathy, and headache.

The exact incidence of LU is unknown, and the average age at diagnosis reported in a large series of patients by Farhi et al. 2 was 16.6 years.

The etiology remains unclear, although infectious or idiopathic causes seem to be associated. Its onset is associated with an exacerbated immune response to viral diseases, such as Epstein-Barr virus, cytomegalovirus, influenza virus, paratyphoid fever, toxoplasmosis, *mycoplasma pneumoniae* , and mumps. However, in most cases, the association with an infection could not be confirmed. Furthemore, these ulcers may as well be caused by drugs.

The exact mechanism involved in the formation of ulcers distant to the primary infection site is poorly understood. It has been suggested the theory of a hypersensitivity reaction to a viral or bacterial infection, leading to deposition of immune complexes in the dermal vessels, which subsequently activates the complement system, resulting in microthrombi formation and consequent tissue necrosis. 2

The ulcers are deep, with red borders and a necrotic centre covered by grey exudate or grey-black eschar, presenting in a mirror pattern. They primarily affect the medial face of the labia minora and vestibule, with variable size, with lesions > 1 cm having been described. Secondary erythema and edema may be impressive.

There is no clear consensus in the literature regarding the precise diagnosis of this pathology. Fahri et al. 2 attemped to establish some diagnostic criteria (major and minor), suggesting that the diagnosis could be achieved if all major and at least one minor criteria were present. The proposed major criteria are age < 20 years, ulcer with sudden onset and acute evolution, first and unique episode, no sexual contact in the 3 months prior to complaint, and absence of immunodeficiency. Moreover, the minor criteria are one or more ulcers with necrotic or fibrinous core, with a well delimited, painful, and symmetrical pattern. Nevertheless, in a retrospective study conducted by Vieira-Baptista et al., 3 the authors concluded that the diagnostic criteria for Lipschutz ulcer should be less strict. 4

Lipschutz ulcer is a diagnosis of exclusion, and it is reached only after precluding other causes of genital ulcer. The differential diagnoses are extensive and include inflammatory processes, drug reactions, trauma, and malignant tumors. Histologic examination is not of diagnostic value because findings are nonspecific. 5 6 7 8 9 10

The treatment is mainly symptomatic. Pain relief is the fundamental aim of supportive care (topical anaesthetics/oral analgesics), even tough, for multiple, large, or deep necrotic ulcerations, topical or a short course of systemic corticosteroids may be considered if the patient fails to respond to topical agents. 11

It is important to emphasize the non-sexual transmission of the disease, and patients should also be informed about the self-limiting nature of the syndrome.

Generally, the natural course is benign, with spontaneous regression within a few weeks.

## Conclusion

The presence of an erosion or ulcer in genitalia usually leads us to think of sexually transmitted infections. However, it is important to have a broader approach and consider non-venereal causes as well. Lipschütz ulcers have been considered an uncommon and probably underdiagnosed entity. Therefore, it is crucial to recognise and include them in the differential diagnosis of vulvar ulcerations; otherwise, patients with this type of lesions will continue to be treated for herpes simplex and other disorders, without any benefit. Besides, this would create unnecessary distress and concern in patients and parents, due to suspected sexual abuse.
